# The Skin Sensitisation of Cosmetic Ingredients: Review of Actual Regulatory Status

**DOI:** 10.3390/toxics11040392

**Published:** 2023-04-21

**Authors:** Iwona Bialas, Sandra Zelent-Kraciuk, Kamil Jurowski

**Affiliations:** 1 CosmetoSAFE Consulting Sp. z o.o., 05-500 Piaseczno, Poland; info@cosmetosafe.pl; 2 The Laboratory of Innovative Research and Analyzes, Institute of Medical Studies, Medical College, Rzeszów University, 35-959 Rzeszow, Poland; 3 Department of Regulatory and Forensic Toxicology, Institute of Medical Expertises, 91-205 Łódź, Poland

**Keywords:** skin sensitization, safety assessment, cosmetic, regulatory status, alternative methods, structure alerts, AOP, IATA, NGRA

## Abstract

All cosmetics products must be safe under foreseeable conditions of use. Allergenic responses are one of the most frequent adverse reactions noted for cosmetics. Thus, the EU cosmetics legislation requires skin sensitisation assessment for all cosmetics ingredients, including the regulated ones (for which the full toxicological dossier needs to be analysed by the Scientific Committee on Consumer Safety (SCCS)) and those (perceived as less toxic) which are assessed by industrial safety assessors. Regardless of who performs the risk assessment, it should be carried out using scientifically and regulatory body-accepted methods. In the EU, reference methods for chemical toxicity testing are defined in the relevant Annexes (VII–X) of the Registration, Evaluation, Authorisation and Restriction of Chemicals (REACH) Regulation. Recommendations for Skin Sensitization (Skin Sens) testing are provided in Annex VII, and this particular endpoint information is required for all EU-registered chemicals. Historically, in vivo animal and human methods have been used. Both raise ethical doubts, and some of them cause practical problems in the objective analysis of skin sensitising potency. Previous decades of huge effort have resulted in the regulatory acceptance of the alternative Skin Sens IATA (Integrated Approaches to Testing and Assessment) and NGRA (Next Generation Risk Assessment). Regardless of the testing issues, a serious sociological problem are observed within the market: the consumer assumes the presence of strong sensitisers in cosmetics formulations and insufficient risk management tools used by the industry. The present review aims to provide an overview of methods for assessing skin sensitisation. Additionally, it aims to answer the following question: what are the most potent skin sensitisers used in cosmetics? The answer considers the mechanistic background along with the actual regulatory status of ingredients and practical examples of responsible industry solutions in the area of risk management.

## 1. Introduction to the Legislative Requirements for Cosmetics in the EU

Each cosmetic, before being placed in an EU market, must be assessed to determine if it is safe under foreseeable conditions of use (art. 3 of Reg. 1223/2009 [1]). This is the obligation of the so-called Responsible Person (RP), i.e., the manufacturer or the importer of the product. If the cosmetic’s ingredients can pose a hazard to human health, its safety must be assessed under foreseeable use conditions. From the point of view of regulatory purposes, if toxicological concerns are serious, they should be evaluated by the Scientific Committee on Consumer Safety (SCCS), an independent advisory body of the regulatory body. SCCS opinions are the basis for ingredient authorisation (i.e., placing the substance in one of the Reg. 1223/2009 annexes). From the industrial point of view, cosmetics ingredients with less toxic or nontoxic effects are not authorised and, due to RP obligations, their safety in intended usage conditions should be as assessed by the industrial safety assessors. For authorisation, the full toxicological dossier of the cosmetics ingredient must be submitted to SCCS. For less toxic substances, as a minimal information, the ingredients’ ability to cause skin and mucus membrane irritation, their sensitisation potential, and their genotoxicity should be addressed [2].

Cosmetics created by downstream users (according to the Registration, Evaluation, Authorisation and Restriction of Chemicals (REACH) Regulation [3]) do not require registration as typical chemical mixtures in the EU. The fact that the final product does not need to be registered is based mainly on the sectoral risk assessment obligation. However, each raw material used in cosmetics must meet the regulatory requirements of REACH and the Classification, Labelling and Packaging (CLP) regulations [4].

The chemical manufacturer or importer must assess the potential risk (to human health and the environment) related to its use. Depending on the tonnage of chemicals placed onto a market, the standard information requirements for the hazard assessment vary significantly: the higher the tonnage, the more detail is needed in the dossier. For chemicals with tonnages > 1 and up to 10 t/year, the same minimum information is required as for non-authorised cosmetics ingredients (with the acute toxicity in some circumstances) [2,5].

What is worth noting is that the tonnage-dependent requirements of the toxicological dossiers are not fully implemented into the cosmetics legislation. If a substance is of high concern, its assessment by the SCCS may be addressed, even if its use is not widespread. This sometimes causes an issue: when the registrants from the chemical legislation’s point of view were not initially obliged to perform a full toxicological analysis on their product, it can be involved with the necessity of preparing a substance in-depth toxicological dossier after its commercialisation as a cosmetics ingredient. The situation is even more complex if the animal testing requirements for cosmetics and chemicals must additionally be considered [6].

## 2. Skin Sensitisation: A Major Endpoint of Cosmetics Adverse Effects

Skin allergy is a common phenomenon in both the occupational and consumer side of exposures. Most consumers declare that they have sensitive skin and, as a result, demand cosmetics with reduced allergenic potential (hypoallergenic claims) or at least with controlled sensitisation properties when the avoidance of allergen content is not possible (such as hair dyes). It is worth noticing that consumers’ perception of hypoallergenic claims (assumed lack of allergy) differs from claim support law’s concept [7,8] and the practical possibilities of allergy avoidance.

Usually, up to 20% of the population is assumed to be affected by allergic contact dermatitis (ACD), a disease involved in the cell-mediated immune response to skin sensitisers [9,10,11,12]. The main treatment to avoid clinical manifestations of ACD is to limit or exclude allergen exposure. From that point of view, a well-defined risk assessment, proper product labelling, usage instructions, and precautions are essential factors for the satisfactory health and welfare of consumers. This is done with the following restrictive measures of the CLP and from cosmetics regulations:

- Banning sensitisers or limiting concentrations for consumer and/or occupational usage;
- Labelling rules for chemicals, including hazard statements and EUH phrases (H317 and EUH 208);
- Art. 19 labelling requirements for cosmetics; additional wording of conditions of use and warnings for sensitizers (Reg. 1223/2009, annex III and V).

From the perspective of cosmetovigilance/post-marketing surveillance, ACD is one of the most frequently observed undesirable effects related to cosmetics use, along with the irritative response (Irritant Contact Dermatitis (ICD)) or complaints in which the precise demarcation between ACD and ICD is difficult. RP statistical analysis of the cosmetics adverse reactions observed are not available to the public; only serious adverse effects should be addressed to the authorities (art. 23 of, Reg. 1223/2009), and problem analysis is limited in the literature. However, some of the data presented confirm general assumptions [13,14,15]. Adverse effects are observed at a noticeable level, but serious allergenic responses are observed: anaphylaxis or death cases are extremely rare. However, their occurrence can be a trigger for regulatory decisions, such as, for example, the request for a comprehensive risk assessment strategy for hair dyes in the EU, which was incorporated in 2004 [16].

## 3. Mechanistic Basis for Skin Sensitization

Skin sensitisers are chemicals that have the intrinsic potential to induce a state of hypersensitivity in humans (or testing animals). The adverse outcome pathway (AOP) has been intensively investigated in the last few decades, and a summary of research was published in the OECD guidance document in 2012 [17].

**Figure 1:**
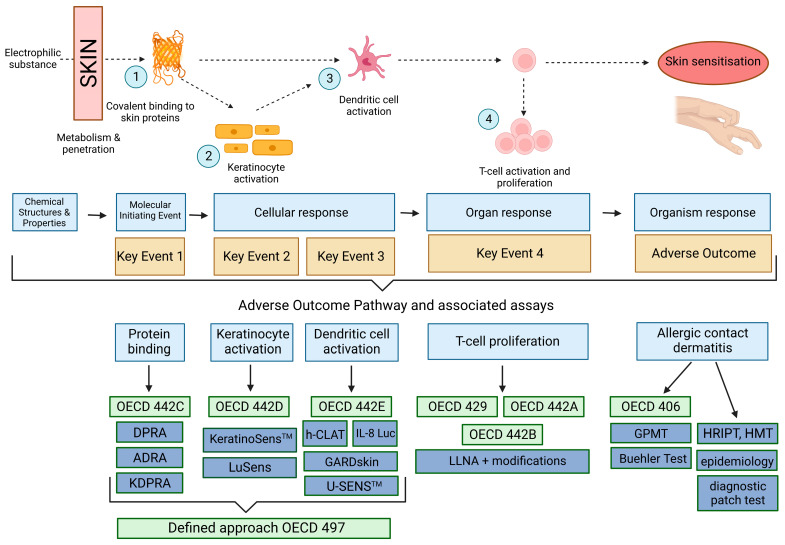
The AOP concept with the main KE and the actual status of validated and scientifically accepted testing methods for skin sensitisation. Created with BioRender.com, accessed on 27 May 2023.

The development of skin sensitisation requires sequential events from the initial exposure of the skin to chemicals, followed by the triggering of the downstream cascade of immune system response, which can be divided into two main steps: the induction (or sensitisation) phase and the elicitation (or challenge) phase. This cascade of events, from the point of view of toxicity pathways, is not as complex as most endpoints of systemic effects. However, the mapping of key events (KE), the definition of its markers, and the development of alternative testing strategies were a great success of the combined cooperation of the cosmetics industry with science. The concept of AOP is presented in Figure 1 with the main KE and the actual status of validated and scientifically accepted testing methods for skin sensitisation.

Induction phase: during initial exposure to chemicals, the specialised immunological memory of this particular substance (allergen) is induced. After its percutaneous penetration, the substance binds covalently to the self-proteins and generates immunogenic neoantigens (MIE). Once the hapten–self-protein adduct is formed, it is recognised by innate immune mechanisms in the skin. This recognition starts with epidermal keratinocyte activation (KE2), where pro-inflammatory mediator secretions and changes in gene expression associated with specific cell-signalling pathways are used as markers of activity. The next steps include the activation (maturation) and mobilisation of Langerhans cells and dermal dendritic cells (DC) (KE3). DCs then migrate to skin-draining lymph nodes, where they present the antigen to naïve T cells and lead to the expansion of educated antigen-specific T cell clones (KE4) throughout the body.

With the elicitation phase, allergic symptoms occur (ACD): the re-exposure to the same allergen activates educated T cells and triggers the inflammatory process responsible for the cutaneous lesions, causing rash, oedema, itchiness, and burning on the exposed skin surface. If the response of the organism is severe, a systemic manifestation can also occur. What is important from the chronic and occupational exposure point of view is that the effects of skin re-exposure usually last a maximum of 2–3 days without further allergen supply, then decline. If skin contact with the allergen is repeatable or prolonged, the clinical manifestation can adversely affect an individual’s health and capacity to perform at work [17,18,19,20,21,22,23].

## 4. Classification Criteria for Skin Sensitisation

Along with the Global Harmonised System (GHS) and CLP criteria, there is only one class of hazard category for skin sensitisers: Skin Sens 1 and the basic hazard testing methods are aimed at defining whether a substance is a sensitiser or not. However, for risk assessment purposes (and practical issues), it is important to discriminate between strong, moderate, and weak sensitisers. It is possible to subcategorise substances on the basis of their sensitisation potency. Potency can be defined as the relative ability of a chemical to induce sensitisation, which is determined by the amount of chemical per unit area required for the acquisition of skin sensitisation in a previously naïve individual. Dose values per unit area can be represented with two metrics of in vivo test results: human no-observed effect levels (NOEL) and animal EC3 values from local lymph node assays (LLNAs). In general, a low dose (EC3: <250 µg/cm^2^) of strong sensitisers is required for sensitisation inductions, whereas weaker sensitisers can require several hundred times higher doses [24,25,26,27]. The subcategories within GHS/CLP distinguish substances within subcategory 1A (strong sensitiser with high potency) and subcategory 1B (sensitiser with low to moderate potency). If the data are not sufficient for subcategorization, the substance should be classified as Skin Sens 1 [4]. Quantitative determination of the potency of skin allergens is critical to assess safe levels of exposure and allow risk management in consumer products.

With the actual state of science, skin sensitisers can be classified based on in vivo, standalone testing methods (however, these data are mainly historical, or this test’s usage is allowed only in some special circumstances with chemical legislation and is forbidden for the cosmetics and its ingredients). For regulatory needs, the tiered or defined approach with in silico/in chemico/in vitro methodologies is accepted as a concept of Next Generation Risk Assessment (NGRA) [28] and Integrated Approaches to Testing and Assessment (IATA), which were published by the Organisation for Economic Co-operation and Development (OECD) [29]. This can be a useful strategy to combine data for substance classification and risk assessment (RA). Most NGRA tests are not standalone methods and can be used only within their applicability domains. Their combination is needed to mimic the cascade of events in the immune response scenario after skin exposure. They should be used with caution, especially for sensitisers that require activation before causing immune events [28,29,30,31].

The first animal in vivo testing protocols for skin sensitisation were used as early as the 1940s (guinea pig tests). Within the next decade, the human repeated insult patch test (HRIPT) was described for the first time. Guinea pig tests were used for a long time for hazard identification purposes (the potency estimate with its use is limited and is based on the frequency of positive responses). The main disadvantage of this method is the decision-making protocol based on the grading scale of the clinical observations.

In the late 1990s, the first mouse LLNA test was validated, and since that time (along with some modifications), until the recent development of alternative testing techniques, it had long been perceived as the gold standard for sensitisation hazard and potency estimation. The obtained EC3 value, used for the potency assessment, is the effective concentration of the test substance required to produce a threefold increase in lymphocyte proliferation compared to vehicle-treated controls (OECD 429) [32].

Methods with keratinocyte activation (KE2) and dendritic cell response (KE3) were adopted in 2018 by the OECD. The validation of the methods that quantify protein binding properties (KE1) had been a long-awaited process, which was finalised by the OECD in 2022. As mentioned previously, these tests are not standalone. Most of them allow only hazard identification; all have some limitations (such as demand for the proper dosage regime to ensure adequate cytotoxic properties, problems with testing substances that are insoluble/nondispersive in test media, multiconstituent substances/mixtures, and some analytical issues regarding the use of luminescence and flow cytometry techniques) [33,34,35]. However, if the testing protocols’ demands are met and the chemicals are in the applicability domain of these methods, then combining the test results with the proper reasoning, together with additional in silico techniques, allows proper hazard and potency characterisation (which was recently accepted with the OECD 497 guideline) [36].

The NGRA concept was also addressed practically for cosmetics purposes. The attempt to confirm regulatory decisions from the past with the new methodology usage was performed and published for INCI (Methyldibromo glutaronitrile (MDBGN; CAS: 35691-65-7)) by Gilmour et al. [28]. The case study concluded that 0.1% MDBGN in a face cream is unsafe (as the estimated exposure level exceeds the calculated safe level of exposure (AEL) for the substance).

In addition, SCCS, with the 11th revision of their guidance notes for risk assessment [2], noticed NGRA as a valuable tool for the analysis of skin sensitisers. The revision was published in 2021; however, since then, only a few cases where the alternative testing for skin sensitisation was used were submitted to the SCCS (SCCS/1645/22: Preliminary Opinion on sodium bromothymol blue [37]; SCCS/1629/21: Final Opinion on Gold (nanoforms used in cosmetics) [38]). It can be simply related to the fact that, up to now, the ingredients assessed by the Committee possess necessary in vivo data or that the NGRA concept has not achieved widespread usage at the moment.

Potency estimation can also be performed with different human data. Sensitiser categorisation is possible with the analysis of historical data on the effective concentration of the induction phase (NOEL) or, more often, the lowest dose tested with no effects (LOEL); additionally, it can also be based on epidemiological data (high number of contact allergies observed in the population), or the high frequency of positive responses in diagnostic patch tests and case studies [39]. These data possess ethical concerns; however, this is an important element of the weight of an evidence-based approach.

In vivo animal data, as well as alternative methods, are not always an accurate predictor of human potency [27]. It needs to be mentioned here that the LLNA EC3 values are used as a surrogate of human potency (as expressed by NOEL). For most tested individuals, the good correlation between human data and EC3 values is obtained. However, some over- and underpredictions were identified. The differences were noted in cases where the difference in species metabolic capability is important. For prehaptens, the difference in human vs. murine test methods (the air oxidation ability in occluded vs. nonoccluded test conditions) and, from the same line of reasoning, the storage conditions can be crucial for the observed potency. The occluded **vs.** non-occluded conditions can also be a simple explanation of observed differences in tests of the volatile compounds. Another factor that needs to be addressed is the problem of impurities where the target compound is weak or a non-sensitiser but its impurities are high-potency chemicals. Last but not least, it must be taken into account that false results can be observed with some “active” skin substances (aside from the mechanism of action), such as LLNA false positive results for sodium lauryl sulphate (SLS), one of the most known surface-active irritants, and anomalous values for salicylates (e.g., hexyl- and benzyl-), in which the inflammatory properties of the substances or its main metabolite (salicylic acid) can be involved with observed EC3 values [40,41].

Those limitations of LLNA testing need to be remembered when the alternative testing methods are analysed. For most of them, the in vivo EC3 values are used as reference for validation.

The available methods for skin sensitisation hazard and potency estimation, along with their regulatory utility, are briefly presented in Table 1 and they are also presented in Figure 1.

## 5. Regulatory Acceptance of Testing Protocols of Skin Sensitisation

Hazard assessment in the EU is presented in Reg. (EC) No. 440/2008, which lays down test methods pursuant to REACH and additionally in REACH Annex VII. The primary version of Annex VII consisted only of information about the LLNA protocol as a gold standard for testing (however, other animal and human in vivo tests were addressed with CLP classification criteria). Annex VII consists of additional recommendations when testing is not mandatory due to the physicochemical properties of the substances (annex VII, point 8.3.). Along with significant scientific progress, the test criteria were first changed in 2017. The amendment assumes that in vivo animal tests can be performed only as a last resort and only if alternative methods are not applicable or the results obtained from those studies are not adequate for classification and risk assessment.

Recently, Reg. No. 440/2008 was also updated [43]. The alternative methods (presented in Figure 1) were added to the list of “international test methods recognised as appropriate for generating information on intrinsic properties of substances”.

REACH requirements allow for animal testing when alternative methodologies are not conclusive, but it needs to be remembered that with the cosmetics legislation (art. 18 of Reg. 1223/2009), it is banned [1]. Both chemical and cosmetics legislations do not accept human predictive testing (induction of sensitisation in healthy volunteers). However, the human repeat insult patch test (HRIPT), along with other patch tests, is routinely performed for cosmetics as a confirmatory test to determine if an ingredient and/or product can be assumed to have good skin tolerance. These test results are not useful for hazard assessment purposes, but they can be an important argument in RA (lack of adverse effects under foreseeable conditions of use) [39].

The statistical analysis of the registration dossiers notified to the ECHA gives a summary that includes the frequency of use of each method and their utility, and it is proof of huge development in skin sensitisation assessments in the last decade. Statistical analysis was performed with QSARToolbox 4.5 SP1 and the ECHA REACH database (updated version 2021) on 13 March 2023. It should be noted that the accuracy of the information in the database was not validated and that a possible read-through of data was not excluded; thus, the numbers presented in Table 2 are for illustrative purposes only. Regardless, if we compare a few decades of history of in vivo testing with the usage of alternative testing methods (acceptable for regulatory purposes since 2017), it is clearly visible how important alternative methods are and how impressive their growing popularity is. For each defined testing category, the number of chemicals tested is presented along with the number of experimental data points obtained.

## 6. Structure–Activity Relationships: Electrophilic Nature and Skin Penetration Ability of Haptens

Skin sensitisation is a result of skin exposure to haptens, specific molecules that can covalently bind to endogenous proteins (as the main proposed mechanism of action). These protein-binding properties result, in general, from the electrophilic nature of the chemicals.

The hapten nature of the chemical can be directly present in the molecule, or it can be induced by its abiotic or metabolic transformations. Prehaptens are pre-electrophilic substances that require oxidation prior to skin contact. Prohaptens are also pre-electrophiles, but they are not protein-reactive unless they are metabolically activated in the skin. The picture is even more complex as a result of the fact that moderate or weak hydrogens can be oxidised or metabolised to stronger derivatives. The other option is that some pre-electrophiles are able to be both abiotically and metabolically modified. In addition, the last scenario should take into consideration the problem of cross-reactivity, in which different chemicals can be involved with the same or similar reactive derivatives [18,44,45,46]. The additional problem of substance purity was presented previously.

The second demand for the chemical to be a skin sensitizer is its capability to cross the epidermal barrier. Although structure–skin penetration relationships have been analysed for several decades, there are no scientifically acceptable in silico prediction techniques that can be used as the gold standard in a wide range of different structure chemicals [47,48,49]. In general, low-molecular weight substances (MW < 500 Da) with moderate lipophilicity (logP = −1 ÷ 4) and a nonionic character are favourable for epidermal transport. If a precise estimate of percutaneous penetration is needed, it can be performed with validated methods (in vitro OECD 428 is preferable for cosmetics).

The defined natures of the chemicals (protein reactivity and ability to cross the epidermal barrier) are described as molecular initiating events (MIE) in the concept of AOP skin sensitisation. Reactive electrophilic centres present in chemical structures will result in covalent protein-binding properties. Those mechanistic domains identified in the allergens were intensively analysed in the past, resulting in five common mechanistic domains defined for Skin Sens structure–activity relationships (SAR). All of them are presented in Table 3 and are the basis for in silico chemical profiling for skin sensitisation. The recent progress and the rule-based method of sensitiser categorisation resulted in the generation of one more category: substances with a lack of structural alerts that are, regardless, prone to oxidation and the generation of reactive hydroperoxides [50,51].

The mechanistic domains are not synonyms for chemical classes. Chemicals from different classes can directly react along with the defined mechanistic domain identified in the structure, and different-class chemicals can even be modified to have structures with the same mechanistic domain.

This structure-based or mechanistic domain-based analysis is not complicated for simple chemicals. It can be performed with some expert rules or with in silico profiling software (some of the commercially available possibilities are freely assessable (such as QSAR Toolbox developed with OECD and ECHA cooperation), whereas others require a licence (DEREK Nexus)) [28,36,52]. In silico profiling is a fast and easy screening technique for potential sensitisers. It can even be performed by users without advanced knowledge (e.g., the QSARToolbox offers the automated workflow option, in which the system will perform the process without manual step control) [53]. However, it must be noted that those in silico predictions are theoretical considerations based on gathered knowledge. Sometimes, decisions may be difficult without confirmatory testing. In a real-life situation, some substance transformations are more favourable; it depends on the environmental conditions (solvent type, media pH, oxygen content, coreactants, etc.). Even if, up to now, there are some algorithms used to generate plausible metabolic maps (such as the OASIS TIMES (TIssue MEtabolism Simulator) model for skin sensitisation) and the incorporation of in silico analysis is accepted as part of the ITS v1 or v2 Defined Approach methodology (OECD 497), the results always need a weight of evidence approach. If the structure is complex (more reactive functionalities are present in the chemicals)–the in silico profiling can give biases. In such cases, *in chemico,* in vitro tests of protein binding properties should be performed as the best supporting choice.

## 7. Risk Assessment of Skin Sensitisers

The RA of skin sensitisers has long been problematic; it is (especially by the non-experts) believed that allergies can be observed independently from allergen exposure. However, based on the knowledge about the AOP of sensitisation, the thresholds for the induction and elicitation phases can be estimated, at least theoretically. For risk management purposes, it is essential to ensure that consumers and workers during their daily activities are not exposed to sensitisers at levels that allow induction of the immune response (primary prevention of allergy). Second, there is a need to eliminate or reduce elicitation reactions (secondary prevention) for individuals who are already sensitised (this is routinely done by regulatory bodies, including risk management and communication).

From the primary prevention point of view, similar to the systemic effects, the point-of-departure (PoD) values can be estimated. The concept of the dose of a sensitiser not expected to cause sensitisation induction (No Expected Sensitising Induction Level (NESIL)) was developed for fragrances as part of the Quantitative Risk Assessment (QRA) methodology (and within an actual revision as QRA2) [54,55,56], and it is successfully used for the risk assessment of fragrances within industries working in agreement with so-called IFRA Standards. The estimation of NESIL values requires LLNA (EC3 values) and/or human test results (LOEL and NOEL values). With NESIL, after the addition of selected sensitisation assessment factors, the acceptable exposure level (AEL) is derived and combined with the actual exposure to the sensitiser contained in the particular product type. This approach is routinely used within industrial safety assessments. In the cosmetics industry, the QRA assessment is performed by the fragrance materials suppliers due to the fact that the exact raw material compositions are usually not presented to downstream users (regardless of the origin of the mixture (synthetic vs. natural) or due to trade secret issues).

The QRA methodology is widely used for fragrances; however, its main disadvantage comes from the necessity to use in vivo data for NESIL (PoD) estimates. If in vivo data are not available, the safety assessment of the substance (in light of the animal test ban for cosmetics) is a huge practical problem.

Recent advancements in alternative methods could be a chance for EC3 prediction based on the AOP skin sensitisation KE1–KE3 (details in Figure 1) [57]. Natch and Gerberick [58] compared the predicted PoD values with historical data, and the comparison seems to be very promising. The use of regression models with the results of the k-DPRA, KeratinoSens, and h-CLAT tests for EC3 prediction could be a chance for safety assessors to use uniform PoD estimation and fill the data gaps for existing sensitisers and the future innovations in that field.

If we assume that there is a dose–response threshold for skin sensitisation, then the next step, the exposure-waiving in RA, is a natural consequence of the scientific acceptance of the fact. Similar to the systemic effects concept, the threshold of toxicological concern (TTC) [47], a very low level of skin exposure can be determined, below which there is no appreciable risk of sensitisation. Such assumptions can be very useful in cases where the skin sensitisation potential of the substance is not known, but the negligible exposure can justify the decision not to conduct the tests. The concept, called the dermal sensitisation threshold (DST), was first published by Stafford et al. in 2011 with a DST value of 900 μg/cm^2^ for nonreactive chemicals [59] based on a LLNA dataset. The concept of DST was next extended with protein-reactive chemicals with a value of 64 μg/cm^2^ based on 233 chemicals [50]. Additionally, similar to the TTC rule-based approach, a set of structural rules was also published to identify any chemicals within the High Potency Category (HPC) that were likely to have EC3 values below reactive DST [40]. A third DST value of 1.5 μg/cm^2^ was published, covering those chemicals predicted to be HPC [60]. The recent development presented by the Lhasa company incorporates the DST approach to in silico prediction of chemicals with Derek Nexus software [51].

The negligible exposure reasoning with DST, similar to TTC, could be a first step of skin sensitisation NGRA. The possible acceptance of the DST concept by the SCCS for cosmetics purposes could solve a common practical problem for data-poor cosmetics chemicals. Similar to the TTC concept, it has a finite applicability domain, and it is not useful for mixtures with unknown sensitisation potential.

A successful primary prevention strategy, as well usage of QRA and DST concepts, can only be done with a precise a real-life exposure assessment. Thus, a lot of work is done in that area as a critical step in risk assessment (e.g., occupational hairdresser exposure estimation, the analysis of the cosmetics usage patterns [59], as well as a probabilistic approach for exposure to fragrances [60,61,62]).

## 8. Regulatory Status of Skin Sensitisers in Cosmetics

Cosmetics, as complex mixtures, can be involved with significant sensitisation risk. There are some special products categories in which the presence of skin sensitisers should be avoided (such as hypoallergenic products, cosmetics for pregnant women, and cosmetics for sensitised or atopic skin; in addition, products for babies < 3 years old should be composed of ingredients of low/lack of sensitising properties). On the other hand, there are cosmetics categories in which there is a significant risk. The greatest example is hair dyes, but it also includes perfumery products and cosmetics with high amounts of natural ingredients, especially with high essential oil content.

When considering the toxicological nature of cosmetics ingredients, we always have to bear in mind the horizontal chemical legislation status of the used substances. Are there a lot of known sensitisers used in cosmetics? Are cosmetics the main source of sensitisers for consumers?

There are also many myths associated with cosmetics allergies. Some of the sensitisers used in cosmetics are also important for other industries in which the perception of consumers is not as strict. On the other hand, personal hypersensitivity is often misconstrued with the sensitising nature of chemicals. In general, fragrances, preservatives, and colourants are perceived as the main cosmetics sensitisers.

## 9. Risk Management and Communication

For the purposes of risk management (along with other reasons), cosmetics products should list their intentionally added ingredients on the packaging (art. 19 Reg. 1223/2009) [1]. In 1999, on the basis of the opinion of the SCCNFP (the SCCS predecessor), a pragmatic administrative decision set individual labelling requirements for so-called potential fragrance allergens, including concentration limits of 0.01 and 0.001% for rinse-off and leave-on products, respectively. Those 26 substances are routinely labelled on cosmetics and detergent products (based on their sectoral requirements). The list of allergens (annex III of Reg. 1223/2009) was modified twice: the first was the Hydroxyisohexyl 3-Cyclohexene Carboxaldehyde (HICC) ban in 2017 (the case is presented further in the text), and the second was the Buthylphenyl Methylpropional ban in 2021 (due to its carcinogenic, mutagenic, and reprotoxic (CMR) properties). Both allergens are actually placed in annex II of Reg. 1223/2009.

In 2012, the updated opinion of the SCCS (SCCS/1459/11) on fragrance allergens in cosmetics products was published. After thorough analysis with human clinical and epidemiological data as well as animal testing results and structure–activity relationship (SAR) analysis, the SCCS listed established contact allergens in humans, established contact allergens in animals, likely contact allergens, and possible contact allergens. In light of the SCCS opinion, the additional individual labelling requirements were proposed:

- An additional 56 entries should be added to the Reg. 1223/2009, annex III;
- In general, 27 entries of individual labelling should be incorporated for plant essential oils or extracts; however, one entry can constitute a few species from a defined fragrance category, such as Lavandula: hybrida, intermedia, angustifolia or Rosa: damascena, alba, canina, centifolia, gallica moschata, rugosa;
- Some of the existing entries in annex III should be modified (for some ingredients, only the peroxide value limits were allocated; with the amendment, its individual labelling should complement the restrictions).

The proposed date of adoption of the new regulation is expected to be in the first half of 2023.

If the sensitisation potential of the cosmetics ingredient is inevitably connected to its nature and it is a necessary component of the final products, special words (“contains X”), instructions for use, and precautions must be used. A great example of these obligations is the labelling requirements for hair dying products (presented in detail in annex III of Reg. 1223/2009).

Recently, similar formulation recommendations for formaldehyde releasers used in cosmetics have been incorporated. Formaldehyde, after its EU-harmonised CMR classification in 2015, is forbidden for cosmetics usage. However, formaldehyde releasers as preservatives are still allowed for use (authorized with the Reg. 1223/2009, annex V). If these preservatives are used and the free formaldehyde content in ready-to-use formulations exceeds the 0.001% (10 ppm) threshold, the wording “release formaldehyde” must be used on the final product label [63].

## 10. The Statistical Analysis of the Regulatory Status of Skin Sensitiser

Besides SCCS activity and the need for authorisation of identified skin sensitisers in cosmetics, there are some horizontal requirements, such as the IFRA standards for fragrance materials, as well as the most impactful element: the EU-harmonised classification decisions for chemicals. In Table 4, the actual statuses of skin sensitisers authorised in the EU are presented. The public information on the ECHA notifications performed [64] and the CLP-harmonised classifications [65] up to the 18th Adaptation to Technical Progress (ATP) were compared to the data available in the CosIng database. In conclusion, it can be seen that the number of recorded (INCI) names in the CosIng database is impressive (higher than the number of substances notified to ECHA). However, a >1 tonnage per year requirement is involved with the ECHA notification, and there are also some sectors exempt from registration (e.g., food and pharmaceutical chemicals). Therefore, many chemicals are not “visible” in the ECHA database. Additionally, it is reasonable to assume that 10 to 15% of all records from the Cosing database are of practical importance (a lot of substances are of marginal use in cosmetics). Information about the CLP statuses of harmonised cosmetics ingredients was gathered with an advanced search in the ECHA database (uses and exposure category PC39: Cosmetics, personal care products).

From the chemical EU-harmonised classification statuses, it can be assumed that a very low number of substances used in cosmetics are actually regulated. However, it cannot be forgotten that a significant amount of cosmetics ingredients pose no hazard to human health and as such do not require classification. In the Table 5 the detailed statistic on the skin sensitisers is placed. It is clearly visible that a limited number of chemicals with harmonised (CLP) status is regulated with the cosmetics legislation, from the global number of 1070 records in the annex IV of Reg. No. 1272/2008 with its 18ATP, only 130 (a sum of columns (1) and (2)) are identified as cosmetics ingredients (present in the CosIng database with defined INCI name) and from that number 78 of substances are authorised with Reg. 1223/2009 (column (1)). The numbers compared here are gathered with substance CAS identifiers (from the cosmetics nomenclature point of view, some isomers of chemicals possess the same INCI names and should be perceived as one record in CosIng databse).

It needs to be mentioned that according to the art. 15 of Reg. 1223/2009, if the substance has a harmonised classification as a CMR, it is banned for use in cosmetics. The exemption procedure allows for CMR substance authorisation after a positive SCCS opinion about its safety. This procedure was successful for some chemicals, such as salicylic acid or titanium dioxide (nano). In the sum of 78 authorised skin sensitisers (column (1)), 41 substances are banned for use in cosmetics due to the art. 15 consequences (substances sensitise along with CMR properties and were not or were negatively assessed by the SCCS).

In agreement with the actual coexistence of both the chemical and cosmetics legislation (according to Reg. (EU) 2019/83 amending the annexes to Reg. 1223/2009: “in order to uniformly implement the prohibition of CMR substances within the internal market, to ensure legal certainty, in particular for economic operators and national competent authorities and to ensure a high level of protection of human health, all CMR substances should be included in the list of prohibited substances in Annex II …”. As a result, if the substance is simultaneously a CMR and a skin sensitiser, it can be present in the CosIng database, but without the INCI name (reference to real-life cosmetics usage). Thus, from the large number of almost 1000 skin sensitisers not used in cosmetics (column (3)), if they are CMRs, they are all present in the Reg. 1223/2009, annex II.

Additional information on the authorisation status of cosmetics skin sensitisers and their mechanistic domains is presented in Table 6. From the Skin Sens cat. 1, the most important and/or widely used representatives were chosen. The data was compiled with the QSAR Toolbox 4.5 SP1 skin sensitisation profiling tool (using OASIS profiler) with auto-oxidation and metabolism simulations included. Most of the cosmetics ingredients presented in the table should be classified as High Potency Category Chemicals, in agreement with the DST rule-based criteria (presented previously).

From the mechanistic point of view, the most potent sensitisers: protein derivatisation agents, direct Michael acceptors, if only are important for cosmetics purposes, are authorised. Additional sensitisers, with high potency and extensive evidence of contact allergy (from diagnostic tests and epidemiological data)–like thiazolinone derivatives and hair dyes, HICC, were intensively investigated and are also precisely authorised for cosmetic use.

Only five skin sensitisers in cat. 1A are authorised within cosmetics legislation: Isoeugenol, two thiazolinone preservatives, glutaral, and HICC. Maleic anhydride and Bis-Trimethylbenzoyl phosphine oxide are of minor importance if they are present in the formulations but are not intentionally added compounds.

The regulated weak-to-moderate (1B) skin sensitisers consist of commonly used limonene and linalool isomers, methyl- and benzyl salicylates, and two preservatives of minor actual importance: p-chloro-m-cresol and Polyaminopropyl biguanide. Four substances are present in annex II due to their CMR properties. The remaining 11 regulated chemicals (CLP) are not important for cosmetics purposes.

In the most represented category (Skin. Sens. 1), almost 100 substances have potential cosmetics usage (Cosing); however, half of them are not authorised under Reg. 1223/2009 (i.e., they have a minor importance for the industry). In the group of the 48 authorised ones, an important part of them is comprised of hair dyes that all have a quinoid-type structure. P-phenylenediamine, p-aminophenol, and 4,5-diamino pyrazole derivatives are the most commonly used oxidative hair colourant precursors; all require activation (oxidation step), and then they are coupled with colour modifiers to build high-molecular weight condensation products. The SCCS in Memorandum (SCCS/1509/13) considers these structures to be extreme or strong sensitisers based on diagnostic and epidemiological data, and this conclusion is still valid, as allergies to hair dyes are still one of the most noted in society [66]. However, the risk assessment of hair dyes requires thorough exposure assessment, as those products are not intended to be applied on a daily basis, and the presence of couplers in the colouring mixtures can be perceived as a method for fast precursor consumption (and its activated quinoid form) during hair treatment. The kinetics of the reactions were analysed, and it was concluded that the potent sensitising precursors and their self-reaction polycondensates (such as the Bandrowski base for p-phenylenediamine) are not observed in significant amounts in the dye mixture during hair treatment [67,68].

For the purpose of risk management, special instructions of use as well as an allergy test prior to the hair dyeing procedure are recommended for consumers; additionally, wearing protective clothes and gloves is recommended for occupational exposure. The severity of adverse reactions to hair dye precursors is sometimes potentialized by prior exposure to tattoos or false henna tattoo treatment (where higher dermal bioavailability can be observed, and thus, a higher risk of allergy induction needs to be considered) [69,70,71,72,73,74].

The other important part of category 1 representatives includes chemicals that are not intentionally added to cosmetics ingredients but whose traces in the formulation need to be addressed in risk assessment. These include formaldehyde, hydroquinone, acrylamide, acrylate, glyoxal, glutaral, dimethylaminopropylamine (CAS: 109-55-7), which are important residual monomers, amounts of residual substrates, or by-products in the raw materials. The content of these allergens is usually monitored, and the best raw materials, from the point of view of quality and the technical unavoidability of prohibited substances, are chosen routinely in the final product development process.

The next important group of cosmetics allergens includes different chemicals that are prone to auto-oxidation. The classical examples of such structures in Table 5 are limonene and linalool. In practise, it can be a pure terpene, or it can occur in large amounts in natural fragrance mixtures. In a regulatory context, the prevention of auto-oxidation for this type of ingredient is carried out with the quality standards assigned to these ingredients by the IFRA Standards and/or cosmetics regulation. These materials require control of peroxide values [75,76]. For example, the essential oils from the pinus, thuja, and abies families are placed in annex III or in Reg. 1223/2009 with defined peroxide number values. In addition, the materials and final products prone to auto-oxidation are routinely stabilised with antioxidants, and proper packaging and storage conditions should be implemented for these chemical mixtures and should be an important element of safety assessment. The influence of auto-oxidation prevention on health risk was described with the SCCS opinion on fragrances [77] in the cases of limonene, linalool, geraniol, and lavender oil. The EC3 values for pure substances were several times higher than for their “matured” (oxidated with air) analogues.

There are rare cases of a cosmetics usage ban for substances with serious skin-sensitising properties. Regulation decisions are always a consequence of the collection of diagnostic patch tests or epidemiological data.

The first substance, methyldibromo glutaronitrile (MDBGN), was introduced into the market in the 1990s as a promising preservative; however, its toxicological dossier was not fully complemented (the skin-sensitising in vivo data for the substance are lacking). Soon after, the problem of substance ACD became epidemic. Thus, in 2005, the European Commission banned MDBGN from being used in leave-on cosmetics, and in 2008, it was banned from rinse-off cosmetics. A recent case study with NGRA methodology confirms both the utility of the approach for cosmetics purposes and also that the regulatory measure was right [28].

After well-documented case reports, the next ban was introduced in 2013 for vitamin K1 (INCI: Phytomenadione) with the amendment of the cosmetics legislation (Reg. (EU) No 344/2013). The SCCS concluded their opinion about the substance with: “in cases of pre-existing sensitisation acquired by topical application of Vitamin K1 present in cosmetics, an individual might not be able to receive Vitamin K1 therapeutically or experience allergic reactions upon Vitamin K1 treatment”.

The next important allergen banned for use in the EU is hydroxyisohexyl 3-cyclohexene carboxaldehyde (HICC) (typically it is a mixture of two isomers, with the most known commercial name being Lyral). The substance was very popular in floral fragrance compositions; since 1998, it was one of the 26 allergens with an individual labelling requirement in accordance with Reg. 1223/2009, annex III. SCCS, with the opinion SCCS/1456/11, concluded that the number of documented HICC allergies is exceptionally high and recommended a ban on its use, which was implemented in 2017 (Reg. (EC) 2017/141) [78]. With this regulation, the ban was also implemented for two further allergens: chloroatranol and atranol, the main allergenic constituents of Oak moss (*Evernia prunastri*) and Tree moss (*Evernia furfuracea*). Both atranols are not fragrance ingredients per se, but they occur naturally in fragrance mosses. Both are aromatic carbonyl compounds able to form Schiff bases; additionally, for both of them, further pre-/pro-activation to hydroperoxides and Michael acceptor forms is possible. High frequency of contact allergy to *E. prunastri* and *E. furfuracea* noted in eczema patients was addressed mainly to the atranol content in the natural materials. The SCCS concluded that the presence of the two constituents chloroatranol and atranol in cosmetics products is not safe, and as a consequence, they were banned. What is more, the IFRA Standards also restrict the levels of atranol and chloroatranol to a maximum 100 ppm in the mosses. Thus, the tree moss and oak moss available on the market now have the reduced (and controlled) levels of antranols.

Another example of the influence of epidemiology on the authorisation of cosmetics ingredients is the case of triazolinones. The thiazolinones are a group of chemicals with excellent antimicrobial efficacy, which can be obtained with a low effective concentration in formulations. In the late 2000s, after the formation of chemophobic attitudes toward parabens in society, the massive replacement of parabens with triazolinones was observed. The decisions, from the economic and antimicrobial efficacy points of view, are understandable. However, the toxicological dossiers were not considered with caution. The higher popularity of the preservatives resulted in a higher number of allergy cases. The situation was so serious that methylisothiazolinone (MIT) became Allergen of the Year 2013 in the United States [79]. This caused stricter thiazolinone restrictions. SCCS recommends the use of MIT only for rinse-off products; in addition, the maximum concentration of the mixture of thiazolinones (MIT + Methylchloroisothiazolinone) allowed in rinse-off products was significantly reduced.

## 11. Conclusions

Skin sensitisation is an important endpoint for cosmetics risk assessment considering both the nature of the used ingredients and the post-marketing surveillance point of view. The necessary regulatory measures for the prevention of induction as well as the elicitation of allergies are important issues addressed in several regulatory demands and in common market practises. The coexistence of regulatory needs and recent developments in skin sensitisation testing protocols for chemicals, fragrance materials, and cosmetics allows for proper hazard and potency estimation and risk management without the usage of in vivo methods with ethical concerns. As noted in the past, problems with poor data chemicals will probably be solved with a recently validated alternative approach. The importance of the new approach is clearly visible for the requirements of the chemical legislation, but its usage in the case of the cosmetics ingredient risk assessment is limited (which is well-represented by the scientific progress in the literature; however, there are only a few cases of its usage for SCCS toxicological dossier submissions).

The construction of cosmetics legislation ensures high-level protection of consumers from skin sensitisation. If the chemicals are classified as skin sensitisers within CLP legislation, the market monitors show concerns, or the sensitisers are considered important for the EU industry, then the adequate regulatory measure is introduced, even when the impact of the restriction can be demanding (as it is assumed to be for new fragrance materials’ individual labelling requirements and because it was difficult to manage in the past for formaldehyde, HICC, and thiazolinones). There is still demand for a mixture risk assessment methodology; the alternative tests are not suitable for multicomponent ingredients, and this is its main disadvantage compared to in vivo elicitation phase observations. From a practical point of view, great progress can be achieved with scientific acceptance of the threshold nature of skin sensitisation and the usage of DST.

## Figures and Tables

**Table 1:** Skin sensitization—sources of information for regulatory decision making [3,42].

OECD Test Guideline	Latest Update	AOP Key Event Measured	Test Method	Validation and Regulatory Status	Standalone Method	Outcome According to the Test Method/Guideline	Cosmetics Acceptance (EU)
OECD TG 442C	2022	Key Event 1 (peptide/protein binding)	DPRA	+	no	SS or NS	yes
			ADRA	+	no		
			KDPRA	+	no	distinguish Cat 1A from Cat 1B/NS	
OECD TG 442D	2022	Key Event 2 (Keratinocyte activation)	KeratinoSens™	+	no	SS or NS with complementary information	
			LuSens	Validated/under regulatory review	no		
OECD TG 442E	2022	Key Event 3 (Monocytic/dendritic cell response)	h-CLAT	+	no	SS or NS with complementary information	
			U-SENS	+	no		
			IL-8 Luc	+	no		
			GARD™skin	+	no		
OECD TG 497	2021	Defined approach	2 out of 3	+	N/A	SS or NS	
			ITS v1 or v2	+	N/A	NS or SS with potency subcategorization	
OECD TG 429	2010	Key Event 4 (T cell proliferation)	LLNA	validated, accepted as a last resort under REACH/CLP	yes	SS or NS with potency subcategorization (EC3)	only historical data
OECD TG 442A	2010		LLNA: DA				
OECD TG 442B	2018		LLNA: BrdU-ELISA				
OECD TG 406	2022	adverse outcome (clinical manifestation)	GPMT		yes	SS or NS	
	2022		Buehler Test				
-	-	induction phase and clinical manifestation	HRIPT, HMT	not accepted, but can be used as supportive data	yes	SS or NS with potency subcategorization	
-	-	adverse outcome (clinical manifestation)	epidemiology		N/A	SS or NS monitoring of individual hypersensitivity	supportive data for regulatory decisions
-	-		diagnostic patch testing		N/A		

Abbreviations: SS = skin sensitiser, NS = nonsensitiser, Cat 1A = extreme/strong sensitiser according to CLP, Cat 1B = moderate sensitiser according to CLP. The compilation of the methods was prepared based on skin sensitisation: REACH test guidance and OECD test protocols.

**Table 2:** The usage frequency of skin sensitisation test methods for REACH registration purposes (2023).

	Method Type	No. of Chemicals	No. of Data Points
number of chemicals in the database	-	24,123	N/A
chemicals with defined sensitization endpoint	-	8932	33,005
	in chemico	662	1998
	(DPRA)	551	1557
	in vitro	974	5604
	(activation of dendritic cells)	399	1588
	(activation of keratinocytes)	770	2704
	in vivo	8052	24,818
	(LLNA)	4130	15,128
	(GPMT)	3165	2083
	(Buehler Test)	1169	2601
	undefined type of method	387	5785

**Table 3:** The mechanistic domains for skin sensitization with cosmetics ingredient categorization examples.

Michael Acceptors (MA)	Acylating Agents	Schiff Base Formers	S_N_Ar Electrophiles	S_N_1/S_N_2 Electrophiles	Hydroperoxides
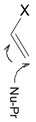	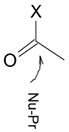	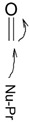	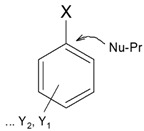	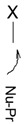	ROOH *
direct MA: p-Benzoquinone Hexyl cinnamal pre/pro MA: Hydroquinoine p-Phenylene-diamine Isoeugenol	Benzoyl Chloride Benzoyl Peroxide	Formaldehyde Glutaral Citral Hydroxycitronellal	-	Methylisothiazoli-none	Limonene Linalool

* No alerts are present in the structure, but autoxidation results in hydroperoxide-active products.

**Table 4:** Skin sensitiser regulatory statuses from a chemical and cosmetics legislation perspective (the date when the detailed information was gathered is presented in brackets).

	Number of Substances	Ref.
REACH Registration statistics (28/02/2023)	22,331	ECHA
CosIng Database (28/02/2023)	30,067	CosIng
Number of cosmetics ingredients with harmonized CLP classification (15/09/2022)	267	ECHA

**Table 5:** Skin sensitizers regulatory status from chemical and cosmetics legislation perspective. Details on the Skin Sens. CLP sub-categorization and cosmetics authorization.

CLP Classification	No of Substances			
	REACH (ECHA)	Reg. 1223/2009		Not Used in Cosmetics (3)
		Authorized (1)	Non-Authorized (2)	
Skin Sens. 1	1020	48, with 26 banned as CMR	52	920
Skin Sens. 1A	29	17, with 11 banned as CMR	-	12
Skin Sens. 1B	24	13, with 4 banned as CMR	-	11

Skin sensitizers regulatory status (EU CLP) and the cosmetics authorization (Reg. 1223/2009).

**Table 6:** Selected regulated cosmetics skin sensitisers along with their mechanistic domains.

INCI Name	Cat.	Reg. 1223/2009, Annex	Mechanistic Domain		
			Hapten	Skin Metabolism	Auto-Oxidation
ISOEUGENOL	1A	III/73	−	MA 1; Schiff BF	MA 1
METHYLISOTHIAZOLINONE	1A	V/57	SN2; SNVinyl	+	−
METHYLCHLOROISOTHIAZOLINONE and METHYLISOTHIAZOLINONE	1A	V/39			
GLUTARAL	1A	V/48	PDA; Schiff BF	+	+
MALEIC ANHYDRIDE	1A	−	Acylation	−	−
BIS-TRIMETHYLBENZOYL PHENYLPHOSPHINE OXIDE	1A	−	Schiff BF	+	−
HYDROXYISOHEXYL 3-CYCLOHEXENE CARBOXALDEHYDE	1A	II/1380	Schiff BF	-	+
LINALOOL	1B	III/84	−	−	ROOH
LIMONENE	1B	III/88	−	−	ROOH
P-CHLORO-M-CRESOL	1B	V/24	−		ROOH, MA 1, MA 4
POLYAMINOPROPYL BIGUANIDE	1B	V/28	Guanidines	−	−
METHYL SALICYLATE	1B	III/324	−	MA1	−
BENZYL SALICYLATE	1B	III/75	Acylation; SN2	+	−
GLYOXYLIC ACID	1B	-	Schiff BF	−	−
HYDROQUINONE	1	II/1339; III/14	−	MA 1	MA 1
P-PHENYLENEDIAMINE	1	III/8a	−	MA 1	MA 1
TOLUENE-2,5-DIAMINE	1	III/9a	−	MA 1	MA 1
1-HYDROXYETHYL 4,5-DIAMINO PYRAZOLE SULFATE	1	III/273	−	Schiff BF	
P-HYDROXYANISOLE	1	III/95	−	MA 1, Schiff BF	MA 1, MA4, ROOH
ALPHA-TERPINENE	1	III/131	−	SN2	SN2
GERANIOL	1	III/78	−	Schiff BF	ROOH, Schiff BF
CITRAL	1	III/70	Schiff BF	−	+
IODOPROPYNYL BUTYLCARBAMATE	1	V/56	Acylation, SN2	−	+
SODIUM HYDROXYMETHYLGLYCINATE	1	V/51	−	release formaldehyde	−
FORMALDEHYDE	1	II/1577	PDA, Schiff BF	−	−
HEMA	1	III/313	Activated alkyl esters	−	Schiff BF
GLYOXAL	1	III/194	Schiff BF	−	−
ACRYLONITRILE	1	II/682	MA 3	−	−
ACRYLAMIDE	1	II/681	MA 4	−	−
DIBENZOYL PEROXIDE	1	III/94	Acylation	−	−

Schiff BF = Schiff base formation; PDA–protein derivatisation agent; MA 1 = Michael addition on quinoid type compounds; MA 2 = Michael addition on polarised Alkenes: alpha,beta-Unsaturated oximes; MA 3 = Michael addition on conjugated systems with electron withdrawing group: Cyanoalkenes; MA 4 = Michael addition on conjugated systems with electron withdrawing group: alpha,beta-Carbonyl compounds with polarized double bonds; Guanidines = Ionic interaction of substituted guanidines with carboxylated proteins; ROOH = Hydroperoxides.

## Data Availability

Not applicable.

## Abbreviations

Skin Sens—Skin Sensitisation and/or skin sensitiser; SCCS—Scientific Committee on Consumer Safety; RA—risk assessment; IATA—Integrated Approaches to Testing and Assessment; NGRA—Next Generation Risk Assessment; KE—key event; AOP—Adverse Outcome Pathway; ACD—Allergic Contact Dermatitis; ICD—Irritant Contact Dermatitis; LLNA—Local Lymph Node Assay; PoD—Point-of-Departure; NESIL—No Expected Sensitising Induction Level; MDBGN—Methyldibromo glutaronitrile; TTC—Threshold of Toxicological Concern; DST—Dermal Sensitisation Threshold; HICC—Hydroxyisohexyl 3-Cyclohexene Carboxaldehyde; MIT—Methylisothiazolinone.
